# Influence of Air-Barrier and Curing Light Distance on Conversion and Micro-Hardness of Dental Polymeric Materials

**DOI:** 10.3390/polym14245346

**Published:** 2022-12-07

**Authors:** Lucian Toma Ciocan, Elena Iuliana Biru, Vlad Gabriel Vasilescu, Jana Ghitman, Ana-Roxana Stefan, Horia Iovu, Roxana Ilici

**Affiliations:** 1 Department of Prosthetics Technology and Dental Materials, “Carol Davila” University of Medicine and Pharmacy, 050474 Bucharest, Romania; 2 Advanced Polymer Materials Group, University Politehnica of Bucharest, 011061 Bucharest, Romania; 3 Academy of Romanian Scientists, 050094 Bucharest, Romania

**Keywords:** restorative dental materials, crosslinking degree, mechanical properties

## Abstract

This study aims to assess the conversion degree and hardness behavior of two new commercial dental restorative composites that have been submitted to light curing in different environments (air and glycerin, respectively) at various distances from the light source (1 to 5 mm) and to better understand the influence of the preparation conditions of the restorative materials. Through FT-IR spectrometry, the crosslinking degree of the commercial restorative materials have been investigated and different conversion values were obtained (from ~17% to ~90%) but more importantly, it was shown that the polymerization environment exhibits a significant influence on the crosslinking degree of the resin-based composites especially for obtaining degrees of higher polymerization. Additionally, the mechanical properties of the restorative materials were studied using the nanoindentation technique showing that the nano-hardness behavior is strongly influenced not only by the polymerization lamp position, but also by the chemical structure of the materials and polymerization conditions. Thus, the nanoindentation results showed that the highest nano-hardness values (~0.86 GPa) were obtained in the case of the flowable C3 composite that contains BisEMA and UDMA as a polymerizable organic matrix when crosslinked at 1 mm distance from the curing lamp using glycerin as an oxygen-inhibitor layer.

## 1. Introduction

Dental restoration represents the reconstruction of the shape of a broken, fractured or partially damaged tooth using composite or ceramic materials. Although there is a wide range of materials that meet the requirements for dental restorative purposes, modern restorative materials have been constantly submitted to substantial development to improve the mechanical behavior [1,2], biocompatibility [3,4] and the ability to bond [5,6] to the tooth structure. In most cases, nanocomposite formulations are employed based on organic resins such as biphenol-A glycidyl methacrylate (BisGMA) or bisphenol-A dimethacrylate (BisEMA) in combination with other urethane dimethacrylate and inorganic fillers exhibiting different sizes and shapes, such as colloidal silica [7,8], glass ionomers [9] or fibers [10]; these formulations have superior mechanical properties, dimensional stability and improved polish retention compared to typical microfilled composites [11,12,13].

Hardness represents a key aspect in describing and comparing dental restorative composites, being the property that defines the resistance of the material to permanent indentation or penetration [14] and drastically influences the masticatory efforts. The hardness behavior of dental composites has been previously analyzed at microscopic level [15,16]. However, the indentation at nanolevel is considered a precise method to evaluate the hardness–curing depth relationship of a composite from the recorded load-displacement data [16]. Our group previously reported the precise evaluation of nanomechanical properties for PMMA-based dental restorations in relation to the heterogeneity of the materials in the micro-nanoscale level [17]. Both the composite homogeneity and crosslinking degree strongly affect the hardness values of the restorative material.

Modern direct coronal restorative materials are composite materials in which the amount of dispersed hybrid inorganic particles is high, and the polymerization of these restorative resins is carried out by the action of a polymerization lamp with an emission in the range of LED radiation [18,19]. Materials composition and photoinitiator structure and concentration are some of the factors that strongly influence the successful light curing of a resin-based composite material [20]. However, the wavelength and bandwidth of the curing source, as well as the intensity and the irradiation time, strongly influence the performance of the final dental restorative material [21]. The International Organization for Standardization defines the light intensity of curing units as the ISO 4049 standard, which recommends an acceptable intensity for clinical use of 300 mW/cm^2^ with a wavelength range of 400–515 nm [22]. Furthermore, many researchers have studied the influence of the curing time and curing light intensity [23,24,25,26,27,28,29], showing that longer exposure times should be used when resins with small filler nanoparticles are chosen for a restorative treatment; however, the distance between the polymerization source and the material to be polymerized may influence the conversion into crosslinked material as well and implicitly the mechanical properties [30] and longevity of the restorations made of these materials. Clinically, the distance between the light source and the restorative composite is variable depending on the isolation method, usually a rubber dam maintained with clasps is used, on the location of the tooth restored on the arch and the ability of the clinician to get closer with the light source tip to the restoration.

Moreover, it was shown that the light-curing process is also influenced by the environmental conditions so that oxygen radicals inhibit the polymerization process and limit the crosslinking degree of the polymeric restorative matrix [31,32]. Borges and her coworkers recently managed to investigate the influence of glycerin as inhibitor layer over the composite mechanical properties showing that the usage of glycerin increased the crosslinking degree of the final restorative materials compared to air cured composites [31].

Thus, the presence or the absence of an intermediate medium between the source and the sample influences the conversion and implicitly the mechanical properties and longevity of the restorations made from these materials. In this study, four new commercially available resin-based composites from two different manufacturers have been submitted to polymerization under LED radiation considering various distances between the surface of the materials and the light-curing source (1÷5 mm) in different environments (in air and glycerin, respectively) in order to investigate the influence of the light distance on the crosslinking degree of the resin-based composites used as restorative dental materials. Moreover, the aim of the study was to evaluate the nanohardness (*H*) of recently developed dental composites crosslinked either in air or glycerin to better understand the optimum preparation conditions of the investigated commercially available composites for tooth restoration.

## 2. Materials and Methods

In this study two different types of methacrylate resin-based composites provided by two different manufacturers with the same clinical indications were studied and their general characteristics are highlighted in Table 1.

The samples were cut in the form of a disc with the help of the Porcelain Sampler device (device for producing porcelain tabs) standardized for testing dental composite materials [33] and specimens with standard dimensions (10 mm in diameter, 1 mm in thickness, *n* = 3 per composite type) were obtained.

**Figure 1:**
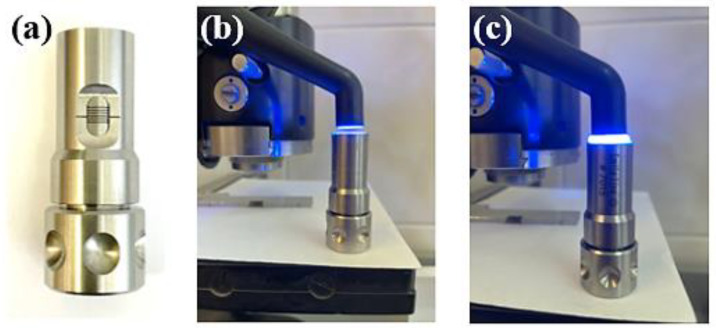
(**a**) The Porcelain Sampler device; (**b**,**c**) the light curing process using Demi Ultra Kit Cordless Light Curing Lamp–Kerr for 20 s.

The polymerization distance calibration was performed with the help of a device specially designed for this research so that the experimental tests were performed in a controlled and repetitive manner to exclude possible measurement errors (Figure 1).

**Figure 2:**
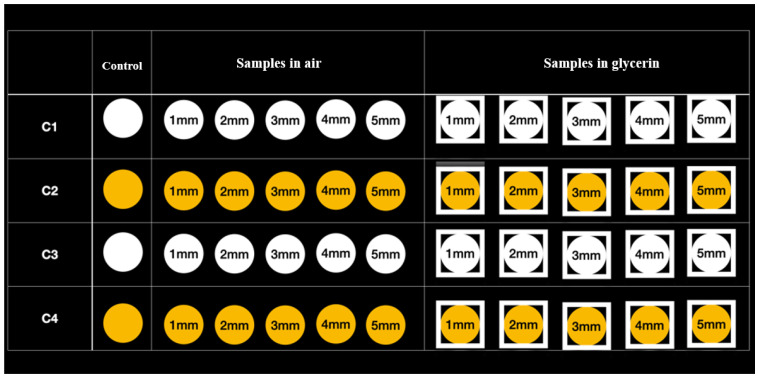
Preparation of C1÷4 dental restorative materials in different crosslinking environments at various distances from the light curing source.

The polymerization process was carried out according to the instructions of the composite manufacturer, using as light source a state-of-the-art LED lamp (Demi Ultra Kit Cordless Light Curing Lamp–Kerr) lasting 20 s for all samples (light intensity: 1200 mW/cm^2^). For each type of restorative material, 3 series of samples were made carrying the polymerization process from the top surface in the open air, respectively in gel with glycerin (air barrier), each sample being polymerized at a variable distance from the source (1 mm, 2 mm, 3 mm, 4 mm and 5 mm) (Figure 2).

The charging dock of the Demi Ultra light-curing unit/Kerr features a built-in, easy-to-use radiometer with LED indicators that instantly communicate the curing light system’s power status. Before each light-curing of every sample, calibration of the light intensity was made using the fully integrated radiometer of the curing unit itself in order to maintain constant light intensity.

### 2.1. Fourier Transform Infrared Spectrometry (FT-IR)

The FT-IR analyses were performed on Bruker VERTEX 70 equipment employing 64 scans in the 600–4000 cm^−1^ range with a resolution of 4 cm^−1^ in total attenuated reflection mode (ATR) by employing a Ge crystal at room temperature.

### 2.2. Nano-Indentation Experiments

The mechanical properties of the investigated restorative materials in terms of micro-hardness (*H*) were analyzed using Nanoindenter G200 equipment (Agilent Technologies, Santa Clara, CA, USA). The samples were mounted and fixed on the Standard stage sample holder and the nanoindentations were carried out employing a triangular pyramid Berkovich diamond indenter with a 20 nm radius. The experiments were performed through Express Test to a Displacement method from the NanoSuit software Version 6.52 (Santa Rosa, CA, USA), accomplishing 400 indents at 50 µm from each other (to prevent interactions between indentations) and Poisson ratio of 0.4 for each sample. In order to minimize the surface effects, as well as to avoid specimen damages and substrate contribution, the displacement into the surface was set to 500 nm [17] and the calculated nano-hardness represents the averaged values over all valid indents performed for each sample. Both nano-hardness properties and conversion degree were measured from the top surface of the cured dental composites.

### 2.3. Statistical Analysis

Statistical analysis of data was performed with commercially available Graph-Pad Prism Software, Version 8.0.1 (San Diego, CA, USA), two-way ANOVA method, considering *p* value < 0.05 as statistically significant. The data were expressed as mean ± SD, *n* = 3.

## 3. Results

### 3.1. Fourier Transform Infrared Spectrometry (FT-IR)

**Figure 3:**
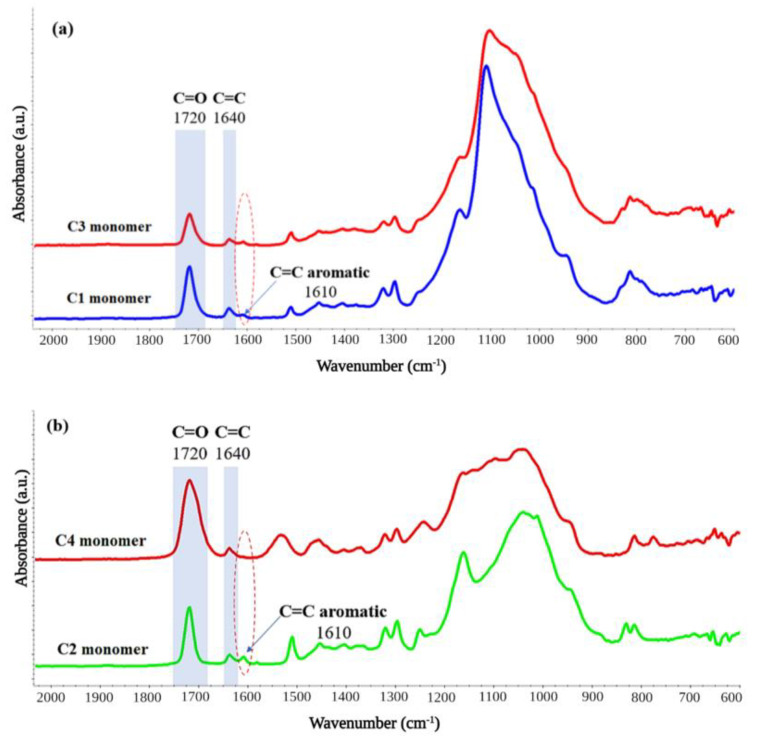
The FT-IR spectra of the (**a**) C1 and C3, and (**b**) C2 and C4 restorative dental materials before the crosslinking process.

All the restorative dental materials (C1÷4) were analyzed through FT-IR spectrometry (Figure 3) before crosslinking under different environments to check their chemical composition; the FT-IR spectra indicated similar methacrylate monomeric structure for all the materials containing the aliphatic C=C signals (~1640 cm^−1^) and the carbonyl C=O (~1720 cm^−1^). Additionally, the presence of the aromatic C=C bonds was observed in the case of C1÷3 restorative materials at ~1610 cm^−1^ as all three investigated materials contain bisphenol A-methacrylate structures in their composition. The signals from ~1400 cm^−1^ and ~1450 cm^−1^ are attributed to the symmetric and asymmetric bending vibrations of the methyl groups and the peaks situated at ~1295 cm^−1^ and 1165 cm^−1^ are assigned to the C-O stretching vibrations of the methacrylate resins. The lack of aromatic groups from the composition of the C4 dental material is clearly observed in the FT-IR spectrum, presenting the characteristic signals of the non-aromatic methacrylate comonomer.

**Figure 4:**
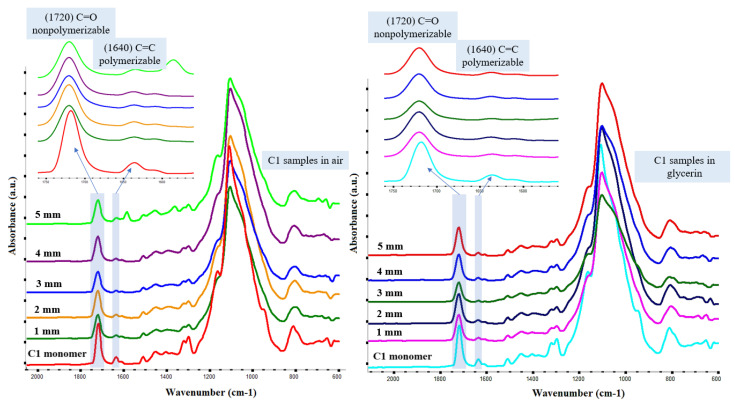
The FT-IR spectra of C1 restorative materials crosslinked in different polymerization environments (air and glycerin, respectively) at various distances from the light curing source.

FT-IR is one of the most used techniques for the conversion degree evaluation in dental materials [34] and the attenuated total reflection (ATR) sampling method was effectively employed as a reliable method for the determination of the nonconverted polymerizable groups from dental composites [35,36,37]. Thus, the degree of nonconverted double carbon bonds (C=C) was determined using the absorbance peak intensities for the C=C bonds situated at ~1640 cm^−1^ (peak attributed to the polymerizable aliphatic chains) before and after curing at different lamp distances (Figure 4).

The conversion degree (CD) into polymer network under LED lamp for each material was calculated according to the conversion of the corresponding area band of the photopolymerizable C=C (aliphatic) group located at ~1640 cm^−1^ in each sample in comparison with the area band of the nonpolymerizable C=O group that is observed in each investigated sample at ~1720 cm^−1^ before and after light crosslinking process, using the following equation [38]:

$$CD\left(\%\right)=\left[1-\frac{\left(\frac{A_{1640}}{A_{1720}}\right)after\;curing}{\left(\frac{A_{1640}}{A_{1720}}\right)before\;curing}\right]\times 100$$

Methacrylate monomers are among the most widely used resins in dental restorations. Although used as a main component in dental formulations, BisGMA monomer tends to induce strong intermolecular hydrogen interactions within the composite leading to high viscosity. Thus, it is predominantly used together with other methacrylate monomers that have a lower viscosity such as UDMA [39], BisEMA [40] or TEGDMA [41] and act as diluents for proper incorporation of the filling agents and stabilizers. However, an important aspect in using these materials is the crosslinking degree after polymerization as low conversion values will lead to the appearance of residual monomers which are not desirable in dental applications and to poor mechanical properties and durability [42]. Moreover, previous reports showed that the curing environment has a strong influence on the crosslinking degree of the methacrylate monomers, and it was demonstrated that the presence of oxygen radicals during the curing reaction limits the conversion degree by forming polymer chains more susceptible to wearing [31].

In this study, the obtained results regarding the crosslinking degree of each polymerized material at different distances from the curing lamp both in air and using glycerin are presented in Table 2.

In the case of the flowable dental restorative material C1, no significant influence of the environment in which the material was subjected to the photopolymerization process is observed. Thus, the crosslinking process of the C1 dental materials in the presence of air proceeded almost similarly as in the case of using glycerin as a covering layer. In the case of C1 samples, it is observed that the distance at which the photopolymerization lamp is located does not significantly influence the crosslinking degree of the methacrylate resins in the range of 1–3 mm. However, by increasing the distance between the surface of the sample and the light-curing lamp, a slight decrease in the crosslinking degree can be observed at 4 mm from the sample, and even more drastically by positioning the lamp at 5 mm from the sample when the photopolymerization process takes place in air (down to ~38%).

For the restorative material C2 found in a more viscous form, the crosslinking medium has a slight influence on the crosslinking degree so that an improvement on the monomer to polymer conversion is observed when glycerin is used as a coating layer. The application of the light-curing lamp at the smallest distance from the sample (1 mm) shows in this case a conversion of the methacrylate structure into crosslinked material which is approximately 23% greater when glycerin is used compared to the oxidative medium.

However, this trend is not maintained when the photopolymerization lamp is positioned at higher distances from the sample, observing an almost similar behavior for both working environments at distances of 2 and 3 mm. By increasing the distance from the lamp, a decrease in the crosslinking degree is observed for sample C2, although maintaining a higher crosslinking degree for the samples prepared by coating with glycerin, which highlights the role of glycerin as a barrier in this case.

In the case of the C3 restorative material, the highest conversions of the flowable methacrylate monomer into polymer are observed, obtaining conversions of up to ~90%. Moreover, even from the minimum polymerization distance (1 mm) it is observed that in the case of this material, higher conversions are obtained (over 55% in air and more than 75% in glycerin). Higher crosslinking conversions when glycerin is used are also obtained in the case of the C3 sample, but with significantly higher values than in the case of the other materials. For the light-crosslinked samples at intermediate distances from the lamp (2 and 3 mm, respectively), no major changes in polymerization conversion are observed, regardless of the crosslinking medium employed. However, increasing the distance between the sample and the LED source to more than 4 mm leads to drastic changes on the degree of polymerization of the C3 material, resulting in a sudden decrease in the crosslinking degree in the case of the oxidative polymerization environment.

In the case of the C4 viscous restorative material, a completely different photopolymerization trend is observed compared to all the other dental restorative materials, including the C3 sample belonging to the same manufacturer. Thus, in the case of the C4 material, the polymerization in an oxidizing environment does not negatively influence the degree of conversion of the monomer into crosslinked material. On the contrary, significantly higher values of the crosslinking degree are observed when the material is not covered with a film of glycerin during polymerization. This fact may be attributed to the absence of aromatic rings in the composition of the C4 sample (as observed from FT-IR spectra, the C4 sample is the only one that does not contain aromatic rings) which could induce steric hindrances in the crosslinking process by hindering the conversion of the C=C polymerizable double bonds into crosslinked chains. In the case of the C4 sample, the lack of aromaticity leads to a greater degree of flexibility in the material, thus allowing easier access to the C=C double bonds during the crosslinking process.

### 3.2. Nano-Indentation Experiments

**Figure 5:**
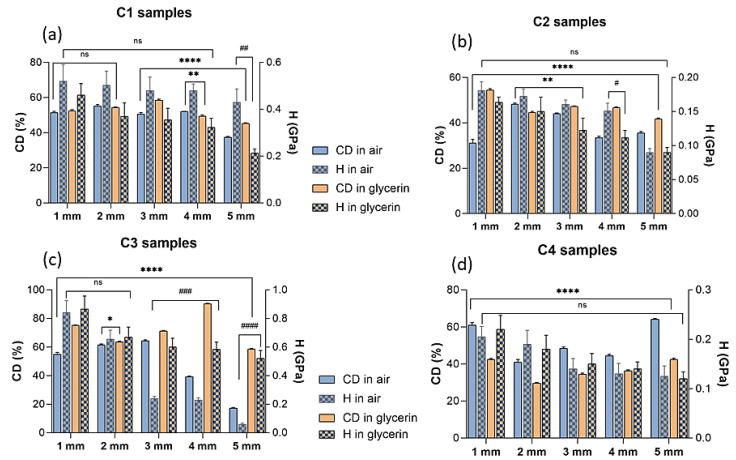
Impact of the light-curing distance on crosslinking degree (statistical significance: ns > 0.5; * *p* < 0.5; ** *p* < 0.005; **** *p* < 0.0001) and hardness (ns ˃ 0.5; # *p* < 0.5; ## *p* < 0.01; ### *p* < 0.001; #### *p* < 0.0001); (**a**–**d**) comparison of the same sample crosslinked in different environments—air and glycerin, at the same depth).

Figure 5 presents the nano-hardness behavior of all the crosslinked dental restorative materials determined by nanoindentation method. The nanomechanical results were studied in correlation with the crosslinking degree values obtained from FT-IR data for each type of dental resin cured in different environments at various distances from the curing lamp. In case of dental restorative materials, the hardness properties are one of the most significant features being considered as an indicator for the endurance of the material to constant indentation or penetration [14]. Analysis of the nanoindentation data showed significant statistical differences among the samples cured under the light source both in air and glycerin environment, respectively, in terms of nano-hardness and crosslinking degree during the irradiation. As expected, Figure 5 reveals that the micromechanical behavior for the light cured samples from 1 to 5 mm levels presents decreasing hardness values for the restorative dental materials as the light curing source is positioned at higher distances from the samples’ surface, regardless of the curing environment. Interestingly, it was noticed that the light curing of C1 and C2 polymeric materials exposed to an oxygen environment leads to a significant improvement of the hardness properties when compared to C3 and C4 samples. However, a drastic decrease in the hardness behavior is observed especially in the case of air-cured C3 samples from 0.84 to 0.06 GPa for the 1 mm to 5 mm levels, respectively, indicating that the oxygen from air may cause an interference in the crosslinking process of the materials, as also suggested from FT-IR data. Moreover, the nanoindentation data showed that although the minimum hardness values were observed at 5 mm light exposure level regardless of the curing environment, the polymeric composites exhibited slightly greater hardness values when they were covered with the glycerin layer to hinder the oxygen radicals‘ involvement in the crosslinking process. Therefore, from all the investigated materials, the maximum value of hardness was obtained in case of C3 glycerin-covered sample (0.86 GPa) exposed at 1 mm from the light curing source and the minimum hardness values were obtained in case of C2 and C3 dental materials prepared in air conditions, showing only 0.06 Gpa and 0.09 Gpa, respectively, in surface hardness values. Additionally, these findings are also in agreement with the results obtained from FT-IR data which revealed that in the case of air curing of C2 and C3 dental composites, the lowest crosslinking degrees are obtained (Table 2).

## 4. Discussion

Commercially available polymer restorative composites are predominantly methacrylate resin-based formulations that typically include BisGMA or BisEMA as main component and TEDGMA and/or UDMA as viscosity regulation co-monomer. Although the monomer mixture is rapidly polymerizable and exhibits good mechanical properties, the linear structure of the less viscous compounds leads to a significant volumetric shrinkage [41] that could affect the long-term clinical behavior of the dental materials generating mechanical fractures and even providing interfacial gaps for bacterial attachment and tooth deterioration [43]. Thus, the incorporation of 60 to 90 wt% of inorganic nano-fillers into the methacrylate matrix is desirable in order to reduce the volume shrinkage upon polymerization and increase the hardness and wear resistance [44] of the dental materials. The inorganic filler content and nanoparticle sizes also influence the monomer-to-polymer conversion degree [45,46], as non-uniform size distribution could lead to lower intermolecular chain interactions and a less compacted arrangement of the polymeric network. Moreover, the presence of the filler can influence the mode of transmitting the curing light in the composite, which in the end will affect the degree of conversion [47]. At the same time, the use of inorganic nano-fillers in resin-based composites has the advantage of a weak interaction with light during the photopolymerization process, due to their small size, which will cause a greater interaction of curing light with the composite material and, respectively, lead to a higher degree of crosslinking [48]. In this study, comparing the polymerization behavior of the BisGMA-based resins produced by the same manufacturer (C1 and C2, in air) but with different viscosity (see Table 1), it was found that the sample with lower viscosity leads to higher crosslinking degrees compared to the more viscous sample (C2), which is expected considering that the flowable sample exhibits higher flexibility degrees of the macromolecular chains; thus, a positive evolution regarding the degree of crosslinking is observed. This trend is also observed in cases involving glycerin as a protective barrier, so that for the more viscous sample, lower degrees of polymerization are obtained (Table 2).

As expected, the incorporation of lower dimensioned inorganic nanoparticles (20–40 nm) in case of C1 and C2 restorative materials allows overall the formation of higher crosslinked polymeric networks compared to C3 and C4 restorative material regardless of the curing environment, even though C1 and C2 exhibit higher inorganic filler content.

The same evolution trend dependent on the viscosity of the samples is obtained for C3 and C4 restorative materials both in air and in glycerin (Table 2) with the observation that in the case of higher distances of the light-curing lamp in an oxidative environment, lower values of the crosslinking degree are obtained in the case of the flowable sample C3, which may involve a more pronounced oxidation of the C3 sample with a proportional hindrance of the crosslinking reaction.

An interesting comparison is obtained in the case of the samples from different manufacturers (C1 and C3) both in air and in glycerin. In the case of these samples with low viscosity, higher degrees of polymerization are obtained for sample C3 both in air and in glycerin, which can be explained by the higher content of polymerizable C=C double bonds of sample C3 compared to C1 (Table 2). In a similar way, by comparing the C2 and C4 materials crosslinked in air, an increase of the polymerization degree is obtained for C4 material, regardless of the distance at which the LED lamp is positioned from the sample (Table 2). Surprisingly, a comparison between C2 and C4 samples in glycerin highlights a decrease in the polymerization degree for the C4 sample compared to the C2 sample, which may be caused by the C4 polymeric structure which contains only UDMA and dimethacrylate monomers. Similar findings were observed for other dental composites where it was shown that TEGDMA-based resins similar to C2 exhibit a higher degree of crosslinking when compared to UDMA-based materials [49]. Although the glycerin barrier offers a favorable environment for the development of polymerization, the degree of polymerization is still reduced for the flowable C4 restorative material (Table 2).

The mechanical properties are crucial in case of dental restorative composites for assessing and predicting their clinical performance and durability [14]. Recently, the nanoindentation method has been extensively used to precisely evaluate the mechanical behavior of polymeric dental composites at the nanoscale level at specific depths for measuring in vitro depth of polymerization [50,51,52,53]. Generally, high values of hardness properties suggest an appropriate polymerization [54] of the dental resin network. Additionally, the hardness properties of dental materials are directly influenced by the crosslinking degree achieved after irradiation exposure [55]. In this study, the mechanical analyses performed by the nanoindentation technique revealed the nano-hardness (*H*) of the samples crosslinked at various distances from the polymerization lamp, showing that all investigated composites were significantly different from each other in terms of hardness in both curing environments (air and glycerin, respectively). In addition, the mechanical performances of the dental materials could also be associated to the amount of filler content. It has been previously stated that besides the polymeric matrix type, the hardness behavior is dependent on the size, shape and distribution of the filling agent within the polymeric matrix as well [56,57,58]. Thus, for the restorative material C1, higher hardness values were obtained in an oxidative environment compared to glycerin, independent of the distance from the polymerization lamp (Figure 5a). This trend was maintained for the same manufacturer (C2, Figure 5b), while for samples C3 and C4 from the other manufacturer, the nano-hardness results showed a different behavior, generally obtaining higher nano-hardness values for the samples in glycerin (Figure 5c,d). Considering that hardness is a manufacturing property and represents the ability of material to resist permanent deformation during application of a load, the degree of crosslinking (which is directly driven by the chemistry of material) may be considered one of the main parameters that impacts this property. The findings in this investigation indicated that the nano-hardness of the crosslinked samples depends both on the distance from the polymerization lamp and on the chemical structure of the sample, while the polymerization method (in air or glycerin) has a limited influence on these type of dental restorative materials. However, there are significant differences between the restorative materials from different manufacturers regarding both crosslinking degree and nano-hardness, in both testing environments. The filler size appears to exhibit only a slight effect on the hardness values of the polymeric restorative composites suggesting that smaller nanofiller sizes tend to provide more predictable mechanical behavior of the tested materials. Thus, the filler content, crosslinking degree or the polymeric matrix chemistry might be responsible for the different outcomes in their clinical behavior and durability.

## 5. Conclusions

In this study, various restorative dental materials were submitted to a light-curing polymerization process and the influence of the distance between the materials’ surface and curing lamp under different environments was investigated. From FT-IR results it was observed that the crosslinking degree is directly dependent on the lamp distance from the sample showing that at lower distances of irradiation higher conversions into crosslinked materials are obtained. Moreover, the polymerization degree depends on the viscosity of the employed material so that for the samples with more flexible structures higher crosslinking degrees are observed. The micro-hardness of the crosslinked materials is generally correlated with their crosslinking degree, the nanoindentation method showing that the hardness behavior of the samples is strongly influenced by the distance from the polymerization lamp, and also by the chemical structure of the materials. Thus, the chemistry of the methacrylate matrix affects the monomer-to-polymer conversion with significant impact on the crosslinking degree and hardness of the final dental materials. From the conducted investigations on the commercial dental restorative composites, it was observed that the flowable C3 composite containing BisEMA and UDMA as polymerizable organic matrix exhibits the highest crosslinking degree and the greatest hardness values when polymerized at 1 mm distance from the curing lamp using glycerin as oxygen-inhibitor layer. By increasing the crosslinking network of the polymeric chains, a better hardness behavior will be achieved. Therefore, the study needs to be extended to various composites from the same class of restorative materials. More physico-mechanical properties should be taken into consideration when selecting a composite material for dental restoration, such as wear resistance and color stability.

## Figures and Tables

**Table 1:** Characteristics of the resin-based composite dental filling materials (Flowable vs. Conventional) included in the study.

Sample Name	C1	C2	C3	C4
**Commercial name/** **Country**	GrandioSO Heavy Flow (Voco, Cuxhaven, Germany)	GrandioSO Universal nano-hybrid (Voco, Cuxhaven, Germany)	G-aenial Universal Injectable (GC, Tokyo, Japan)	G-aenial restorative composite (GC, Tokyo, Japan)
**Viscosity Type**	Flowable Universal Restorative Composite	Conventional Universal Restorative Composite	Flowable Universal Injectable Composite	Conventional Anterior Composite
**Curing Type**	Light-Curing	Light-Curing	Light-Curing	Light-Curing
**Resin components**	Methacrylate matrix (Bis-GMA, TEGDMA, Bis-EMA)	Methacrylate matri (Bis-GMA, TEGDMA)	Methacrylate matrix (Bis-EMA, UDMA)	UDMA, Dimethacrylate co-monomers
**Inorganic filler type**	Nano-hybrid Functionalized SiO2 nanoparticles Glass ceramics particles	Nano-hybrid Functionalized SiO2 nanoparticles	Ultra-fine 150 nm homogeneously dispersed barium-glass fillers Full-Coverage Silane Coating (FSC) technology	MFR Hybrid composite Fluoro-aluminosilicates Pre-polymerised fillers
**Filler loading (% by weight)**	83% *w*/*w*	89% *w*/*w*	69% *w*/*w*	84% *w*/*w*
**Filler particle size range (µm)**	20–40 nm	20–40 nm	150 nm	16–850 nm
**Shade of composites**	A2	A2	A2	A2

**Table 2:** Comparison between the crosslinking degree values for all the restorative materials using both air and glycerin as polymerization medium at different distances from the curing lamp.

Sample Name and Analysis Medium	Mean and Standard Deviation of Crosslinking Degree (%) for All the Dental Restorative Composites According to FT-IR Results							
	C1	C2	C3	C4	C1	C2	C3	C4
	in Air				in Glycerin			
1 mm	51.5 ± 0.6364	31.1 ± 1.5556	55 ± 1.3435	61.1 ± 1.3435	52.5 ± 0.6363	54.5 ± 0.6367	75.3 ± 0.3536	42.4 ± 0.5657
2 mm	55.1 ± 0.8485	48.3 ± 0.4242	61.6 ± 0.6362	41 ± 1.4142	54.4 ± 0.3536	44.6 ± 0.4950	63.6 ± 0.5657	29.6 ± 0.4243
3 mm	50.6 ± 0.8485	44.1 ± 0.2828	64.5 ± 0.6364	48.5 ± 0.7071	58.5 ± 0.6367	47.2 ± 0.2828	71.3 ± 0.4243	34.5 ± 0.6361
4 mm	52.1 ± 0.0707	33.5 ± 0.6363	39.3 ± 0.3536	44.5 ± 0.7069	49.5 ± 0.6359	46.7 ± 0.2836	90.3 ± 0.3536	36.4 ± 0.4950
5 mm	37.35 ± 0.4950	35.6 ± 0.6363	17.3 ± 0.4243	64.3 ± 0.3536	45.2 ± 0.4243	41.8 ± 0.3536	58.6 ± 0.5657	42.5 ± 0.6365

## Data Availability

The data presented in this study are available on request from the corresponding author.
